# Supplementary material for: Role and mechanism of NCAPD3 in promoting malignant behaviors in gastric cancer
Source: Front Pharmacol. 2024 Apr 22;15:1341039. doi: 10.3389/fphar.2024.1341039 (PMC11070777; doi:10.3389/fphar.2024.1341039)
Supplement: Supplementary file 11 [file DataSheet2.ZIP › GSEA/GO_CC/my_analysis.Gsea.1599461408869/GO_MITOCHONDRION.html]

Details for gene set GO\_MITOCHONDRION[GSEA]

|  || Dataset | filtered\_dataset.sample\_info.cls#WT\_versus\_NCAPD3\_MUT |
| Phenotype | sample\_info.cls#WT\_versus\_NCAPD3\_MUT |
| Upregulated in class | WT |
| GeneSet | GO\_MITOCHONDRION |
| Enrichment Score (ES) | 0.2031193 |
| Normalized Enrichment Score (NES) | 1.5357983 |
| Nominal p-value | 0.021186441 |
| FDR q-value | 0.6640937 |
| FWER p-Value | 0.958 |
Table: GSEA Results Summary

  

Fig 1: Enrichment plot: GO\_MITOCHONDRION      
 Profile of the Running ES Score & Positions of GeneSet Members on the Rank Ordered List

  

| SYMBOL | TITLE | RANK IN GENE LIST | RANK METRIC SCORE | RUNNING ES | CORE ENRICHMENT || 1 | 51585 | PCF11 | 3 | 1.212 | 0.0124 | Yes |
| 2 | 64172 | OSGEPL1 | 6 | 1.152 | 0.0248 | Yes |
| 3 | 51312 | SLC25A37 | 12 | 1.077 | 0.0339 | Yes |
| 4 | 55149 | MTPAP | 15 | 1.051 | 0.0451 | Yes |
| 5 | 7052 | TGM2 | 17 | 1.045 | 0.0570 | Yes |
| 6 | 57103 | TIGAR | 37 | 0.963 | 0.0537 | Yes |
| 7 | 140707 | BRI3BP | 40 | 0.947 | 0.0636 | Yes |
| 8 | 115209 | OMA1 | 45 | 0.932 | 0.0718 | Yes |
| 9 | 3954 | LETM1 | 47 | 0.924 | 0.0822 | Yes |
| 10 | 117145 | THEM4 | 65 | 0.860 | 0.0792 | Yes |
| 11 | 55794 | DDX28 | 68 | 0.856 | 0.0880 | Yes |
| 12 | 4204 | MECP2 | 70 | 0.856 | 0.0976 | Yes |
| 13 | 2926 | GRSF1 | 75 | 0.849 | 0.1048 | Yes |
| 14 | 9489 | PGS1 | 84 | 0.820 | 0.1084 | Yes |
| 15 | 55006 | TRMT61B | 101 | 0.782 | 0.1052 | Yes |
| 16 | 129607 | CMPK2 | 124 | 0.779 | 0.0972 | Yes |
| 17 | 55687 | TRMU | 128 | 0.774 | 0.1043 | Yes |
| 18 | 285343 | TCAIM | 134 | 0.765 | 0.1096 | Yes |
| 19 | 92935 | MARS2 | 143 | 0.741 | 0.1123 | Yes |
| 20 | 11162 | NUDT6 | 162 | 0.716 | 0.1067 | Yes |
| 21 | 23590 | PDSS1 | 175 | 0.701 | 0.1057 | Yes |
| 22 | 54516 | MTRF1L | 187 | 0.687 | 0.1054 | Yes |
| 23 | 26278 | SACS | 205 | 0.669 | 0.1000 | Yes |
| 24 | 3303 | HSPA1A | 206 | 0.669 | 0.1082 | Yes |
| 25 | 3304 | HSPA1B | 207 | 0.669 | 0.1163 | Yes |
| 26 | 4836 | NMT1 | 208 | 0.669 | 0.1244 | Yes |
| 27 | 79922 | MRM1 | 209 | 0.669 | 0.1326 | Yes |
| 28 | 171425 | CLYBL | 213 | 0.665 | 0.1383 | Yes |
| 29 | 196294 | IMMP1L | 216 | 0.662 | 0.1447 | Yes |
| 30 | 51250 | C6orf203 | 230 | 0.650 | 0.1423 | Yes |
| 31 | 84816 | RTN4IP1 | 232 | 0.649 | 0.1494 | Yes |
| 32 | 91574 | C12orf65 | 238 | 0.646 | 0.1533 | Yes |
| 33 | 83666 | PARP9 | 258 | 0.624 | 0.1458 | Yes |
| 34 | 219293 | ATAD3C | 260 | 0.623 | 0.1526 | Yes |
| 35 | 87178 | PNPT1 | 263 | 0.622 | 0.1586 | Yes |
| 36 | 25973 | PARS2 | 266 | 0.620 | 0.1646 | Yes |
| 37 | 5728 | PTEN | 285 | 0.606 | 0.1577 | Yes |
| 38 | 81889 | FAHD1 | 299 | 0.592 | 0.1546 | Yes |
| 39 | 36 | ACADSB | 302 | 0.589 | 0.1601 | Yes |
| 40 | 647087 | C7orf73 | 308 | 0.588 | 0.1633 | Yes |
| 41 | 56910 | STARD7 | 335 | 0.573 | 0.1497 | Yes |
| 42 | 92399 | MRRF | 347 | 0.569 | 0.1479 | Yes |
| 43 | 9617 | MTRF1 | 349 | 0.568 | 0.1540 | Yes |
| 44 | 166785 | MMAA | 369 | 0.553 | 0.1457 | Yes |
| 45 | 326625 | MMAB | 371 | 0.552 | 0.1516 | Yes |
| 46 | 102724023 | LOC102724023 | 385 | 0.546 | 0.1479 | Yes |
| 47 | 8209 | C21orf33 | 386 | 0.546 | 0.1546 | Yes |
| 48 | 11019 | LIAS | 409 | 0.533 | 0.1436 | Yes |
| 49 | 63929 | XPNPEP3 | 416 | 0.526 | 0.1452 | Yes |
| 50 | 84274 | COQ5 | 419 | 0.523 | 0.1500 | Yes |
| 51 | 55326 | AGPAT5 | 421 | 0.521 | 0.1555 | Yes |
| 52 | 6256 | RXRA | 439 | 0.509 | 0.1483 | Yes |
| 53 | 54931 | TRMT10C | 446 | 0.507 | 0.1497 | Yes |
| 54 | 25880 | TMEM186 | 462 | 0.498 | 0.1438 | Yes |
| 55 | 637 | BID | 478 | 0.487 | 0.1379 | Yes |
| 56 | 5245 | PHB | 481 | 0.486 | 0.1422 | Yes |
| 57 | 9830 | TRIM14 | 486 | 0.480 | 0.1449 | Yes |
| 58 | 8737 | RIPK1 | 493 | 0.477 | 0.1459 | Yes |
| 59 | 4191 | MDH2 | 494 | 0.476 | 0.1517 | Yes |
| 60 | 6742 | SSBP1 | 501 | 0.472 | 0.1527 | Yes |
| 61 | 57460 | PPM1H | 502 | 0.472 | 0.1584 | Yes |
| 62 | 57506 | MAVS | 507 | 0.470 | 0.1610 | Yes |
| 63 | 5188 | GATB | 512 | 0.469 | 0.1635 | Yes |
| 64 | 617 | BCS1L | 513 | 0.467 | 0.1692 | Yes |
| 65 | 3607 | FOXK2 | 522 | 0.461 | 0.1684 | Yes |
| 66 | 132001 | TAMM41 | 524 | 0.460 | 0.1732 | Yes |
| 67 | 204 | AK2 | 529 | 0.458 | 0.1756 | Yes |
| 68 | 34 | ACADM | 530 | 0.457 | 0.1812 | Yes |
| 69 | 56652 | C10orf2 | 531 | 0.457 | 0.1867 | Yes |
| 70 | 90639 | COX19 | 535 | 0.454 | 0.1899 | Yes |
| 71 | 29090 | TIMM21 | 537 | 0.453 | 0.1946 | Yes |
| 72 | 3609 | ILF3 | 544 | 0.449 | 0.1953 | Yes |
| 73 | 78988 | MRPL57 | 557 | 0.441 | 0.1912 | Yes |
| 74 | 10773 | ZBTB6 | 562 | 0.441 | 0.1933 | Yes |
| 75 | 80775 | TMEM177 | 569 | 0.438 | 0.1939 | Yes |
| 76 | 27349 | MCAT | 574 | 0.436 | 0.1960 | Yes |
| 77 | 55245 | UQCC1 | 580 | 0.434 | 0.1974 | Yes |
| 78 | 26164 | MTG2 | 585 | 0.433 | 0.1995 | Yes |
| 79 | 23786 | BCL2L13 | 588 | 0.432 | 0.2031 | Yes |
| 80 | 55735 | DNAJC11 | 625 | 0.409 | 0.1796 | No |
| 81 | 10975 | UQCR11 | 632 | 0.406 | 0.1797 | No |
| 82 | 219 | ALDH1B1 | 638 | 0.405 | 0.1807 | No |
| 83 | 2021 | ENDOG | 648 | 0.400 | 0.1784 | No |
| 84 | 9949 | AMMECR1 | 650 | 0.400 | 0.1825 | No |
| 85 | 51287 | COA4 | 660 | 0.396 | 0.1802 | No |
| 86 | 55157 | DARS2 | 686 | 0.385 | 0.1651 | No |
| 87 | 10452 | TOMM40 | 696 | 0.380 | 0.1625 | No |
| 88 | 55750 | AGK | 697 | 0.380 | 0.1672 | No |
| 89 | 22877 | MLXIP | 701 | 0.377 | 0.1694 | No |
| 90 | 2932 | GSK3B | 714 | 0.367 | 0.1643 | No |
| 91 | 9131 | AIFM1 | 728 | 0.360 | 0.1584 | No |
| 92 | 6182 | MRPL12 | 729 | 0.359 | 0.1628 | No |
| 93 | 54471 | MIEF1 | 744 | 0.354 | 0.1560 | No |
| 94 | 64087 | MCCC2 | 747 | 0.352 | 0.1587 | No |
| 95 | 8803 | SUCLA2 | 767 | 0.340 | 0.1477 | No |
| 96 | 55178 | RNMTL1 | 771 | 0.337 | 0.1495 | No |
| 97 | 9238 | TBRG4 | 786 | 0.330 | 0.1424 | No |
| 98 | 39 | ACAT2 | 799 | 0.322 | 0.1368 | No |
| 99 | 79590 | MRPL24 | 800 | 0.320 | 0.1407 | No |
| 100 | 29960 | FTSJ2 | 805 | 0.319 | 0.1414 | No |
| 101 | 4285 | MIPEP | 815 | 0.307 | 0.1380 | No |
| 102 | 51373 | MRPS17 | 817 | 0.306 | 0.1409 | No |
| 103 | 516 | ATP5G1 | 826 | 0.301 | 0.1382 | No |
| 104 | 6183 | MRPS12 | 838 | 0.280 | 0.1329 | No |
| 105 | 2747 | GLUD2 | 853 | -0.255 | 0.1249 | No |
| 106 | 2746 | GLUD1 | 854 | -0.255 | 0.1280 | No |
| 107 | 3309 | HSPA5 | 876 | -0.288 | 0.1149 | No |
| 108 | 7296 | TXNRD1 | 877 | -0.289 | 0.1184 | No |
| 109 | 54968 | TMEM70 | 879 | -0.291 | 0.1212 | No |
| 110 | 10797 | MTHFD2 | 911 | -0.338 | 0.1007 | No |
| 111 | 3164 | NR4A1 | 912 | -0.341 | 0.1048 | No |
| 112 | 23710 | GABARAPL1 | 913 | -0.341 | 0.1090 | No |
| 113 | 301 | ANXA1 | 914 | -0.342 | 0.1131 | No |
| 114 | 16 | AARS | 921 | -0.349 | 0.1126 | No |
| 115 | 384 | ARG2 | 945 | -0.366 | 0.0989 | No |
| 116 | 55186 | SLC25A36 | 969 | -0.387 | 0.0853 | No |
| 117 | 5163 | PDK1 | 982 | -0.394 | 0.0806 | No |
| 118 | 25902 | MTHFD1L | 986 | -0.395 | 0.0830 | No |
| 119 | 638 | BIK | 1024 | -0.421 | 0.0588 | No |
| 120 | 10018 | BCL2L11 | 1027 | -0.425 | 0.0624 | No |
| 121 | 23764 | MAFF | 1039 | -0.436 | 0.0590 | No |
| 122 | 7132 | TNFRSF1A | 1055 | -0.449 | 0.0526 | No |
| 123 | 1191 | CLU | 1056 | -0.450 | 0.0580 | No |
| 124 | 1543 | CYP1A1 | 1058 | -0.451 | 0.0627 | No |
| 125 | 5889 | RAD51C | 1059 | -0.451 | 0.0682 | No |
| 126 | 81631 | MAP1LC3B | 1060 | -0.452 | 0.0737 | No |
| 127 | 65981 | CAPRIN2 | 1064 | -0.455 | 0.0769 | No |
| 128 | 84706 | GPT2 | 1076 | -0.460 | 0.0737 | No |
| 129 | 2729 | GCLC | 1123 | -0.497 | 0.0433 | No |
| 130 | 54541 | DDIT4 | 1133 | -0.502 | 0.0423 | No |
| 131 | 11132 | CAPN10 | 1141 | -0.508 | 0.0429 | No |
| 132 | 7444 | VRK2 | 1145 | -0.509 | 0.0467 | No |
| 133 | 92014 | SLC25A51 | 1155 | -0.519 | 0.0459 | No |
| 134 | 4084 | MXD1 | 1167 | -0.535 | 0.0437 | No |
| 135 | 665 | BNIP3L | 1185 | -0.556 | 0.0370 | No |
| 136 | 2043 | EPHA4 | 1228 | -0.602 | 0.0110 | No |
| 137 | 8408 | ULK1 | 1243 | -0.622 | 0.0075 | No |
| 138 | 837 | CASP4 | 1245 | -0.623 | 0.0143 | No |
| 139 | 5106 | PCK2 | 1252 | -0.640 | 0.0173 | No |
| 140 | 157378 | TMEM65 | 1283 | -0.672 | 0.0017 | No |
| 141 | 80201 | HKDC1 | 1289 | -0.681 | 0.0060 | No |
| 142 | 1719 | DHFR | 1298 | -0.700 | 0.0082 | No |
| 143 | 84947 | SERAC1 | 1303 | -0.707 | 0.0136 | No |
| 144 | 5997 | RGS2 | 1336 | -0.770 | -0.0024 | No |
| 145 | 51703 | ACSL5 | 1353 | -0.800 | -0.0054 | No |
| 146 | 255488 | RNF144B | 1362 | -0.834 | -0.0016 | No |
| 147 | 25819 | NOCT | 1374 | -0.891 | 0.0005 | No |
| 148 | 2040 | STOM | 1389 | -0.988 | 0.0015 | No |
| 149 | 3329 | HSPD1 | 1402 | -1.183 | 0.0063 | No |
Table: GSEA details [plain text format]

  

Fig 2: GO\_MITOCHONDRION      
 Blue-Pink O' Gram in the Space of the Analyzed GeneSet

  

Fig 3: GO\_MITOCHONDRION: Random ES distribution      
 Gene set null distribution of ES for **GO\_MITOCHONDRION**
